# Glutaminase - A potential target for cancer treatment

**DOI:** 10.37796/2211-8039.1445

**Published:** 2024-06-01

**Authors:** Josephine Anthony, Sureka Varalakshmi, Ashok Kumar Sekar, Nalini Devarajan, Balamurugan Janakiraman, Rajendran Peramaiyan

**Affiliations:** a Department of Research, Meenakshi Academy of Higher Education and Research (MAHER-Deemed to be University), Chennai 600 078, Tamil Nadu, India; b Centre for Biotechnology, Anna University, Chennai 600 025, Tamil Nadu, India; c SRM College of Physiotherapy, Faculty of Medicine and Health Sciences, SRM Institute of Science and Technology (SRMIST), Kattankulathur, Tamil Nadu 603203, India; d Department of Biological Sciences, College of Science, King Faisal University, Al-Ahsa 31982, Kingdom of Saudi Arabia; e Department of Biochemistry, Centre of Molecular Medicine and Diagnostics (COMManD), Saveetha Dental College & Hospital, Saveetha University, Chennai 600 077, Tamil Nadu, India

**Keywords:** Glutamine, Cancer, Glutaminase inhibitor, Glutaminase, Autophagy, Redox homeostasis

## Abstract

The overexpression of glutaminase is reported to influence cancer growth and metastasis through glutaminolysis. Upregulation of glutamine catabolism is recently recognized as a critical feature of cancer, and cancer cells are observed to reprogram glutamine metabolism to maintain its survival and proliferation. Special focus is given on the glutaminase isoform, GLS1 (kidney type glutaminase), as the other isoform GLS2 (Liver type glutaminase) acts as a tumour suppressor in some conditions. Glutaminolysis linked with autophagy, which is mediated via mTORC1, also serves as a promising target for cancer therapy. Glutamine also plays a vital role in maintaining redox homeostasis. Inhibition of glutaminase aggravates oxidative stress by reducing glutathione level, thus leading to apoptotic-mediated cell death in cancer cells Therefore, inhibiting the glutaminase activity using glutaminase inhibitors such as BPTES, DON, JHU-083, CB-839, compound 968, etc. may answer many intriguing questions behind the uncontrolled proliferation of cancer cells and serve as a prophylactic treatment for cancer. Earlier reports neither discuss nor provide perspectives on exact signaling gene or pathway. Hence, the present review highlights the plausible role of glutaminase in cancer and the current therapeutic approaches and clinical trials to target and inhibit glutaminase enzymes for better cancer treatment.

## 1. Introduction

In cancer conditions, reprogramming of metabolic pathways is an important and critical target for the cancer cells to progress, which end up in need of specific nutrients for further proliferation and metastasis [1]. Glutamine, being one of the abundant free amino acids, has indispensable roles such as a precursor for glutathione synthesis, a precursor for proteins, nucleic acids, and lipids as well as an alternative source of carbon in the Tricarboxylic acid (TCA) cycle. Importantly, the synthesis of proline from glutamine is vital, as proline is a key metabolite during adaptation to hypoxia and exerts a regulatory role in cancer conditions [2–4]. Eagle et al. was the group who first reported that HeLa cells consumed 10 to 100 times more glutamine than any other amino acid, and hence confirmed the association between tumour growth with upregulated glutamine metabolism [5]. Following this report, numerous pieces of evidence document that for proliferation and tumor growth, many cancer cells were observed to depend on the exogenous supply of glutamine [6–8], and therefore upregulated glutaminolysis is reported in many aggressive cancer types including breast cancer [9–13]. Various glutaminase inhibitors such as 6-diazo- 5- oxonorleucine (DON), Bis- 2- (5- phenylacetamido- 1, 3, 4- thiadiazol- 2- yl) ethyl sulfide (BPTES), 5-(3-Bromo-4-(dimethylamino) phenyl)-2,2- dimethy l-2,3,5,6tetrahydobenzo [a] phenanthridine- 4 (1H)-one (Compound 968), JHU-083, CB-839, are in current use to unveil the exact role of glutaminase in several types of cancers. Hence, the present review addresses the recent mechanistic role of glutamine in tumorigenesis and the need for its inhibition for effective outcomes.

### 1.1. Glutaminase and cancer

Glutaminase enzyme catalyzes the conversion of glutamine to glutamate, via, a process called glutaminolysis, which is the rate-limiting step in glutamine metabolism. The key role of glutaminolysis is to supply intermediary metabolites to the TCA cycle for cell growth, such as, glutaminolysis produces glutamate andα-ketoglutarate and replenishes the TCA cycle. Thus, glutaminolysis not only provides intermediates for biosynthetic pathways but also supports energy production. Glutaminase exists in two different isoforms based on the site of secretion, GLS1 (kidney-type glutaminase) and GLS2 (liver-type glutaminase). Compared to GLS2, GLS1 exerts an important role in glutaminolysis and tumorigenesis through overexpression in aggressive cancer types, to meet the high requirement of glutamine by the cancer cells [14]. GLS is the gene generally used to denote GLS1 and the gene coding for the liver-type isoform is called GLS2. Over-expression of GLS1 was also reported predominantly in Triple Negative Breast Cancer (TNBC) compared to other subtypes of breast cancer. Further, via dysregulating the glutaminolysis pathway, GLS1 favours the survival and progression of TNBC cells. Phosphate also induces the activation of GLS1 and 2, wherein a high level of phosphate activates GLS1 and a lower level activates GLS2 [15]. In certain circumstances, GLS2 is often recognized as a tumour suppressor, and therefore, down-regulated GLS2 was observed in many cancer types [16]. Further, it has been documented that overexpressed GLS2 provoked antiproliferative properties by arresting the cell cycle at the G2/M phase [17]. On the contrary, Lukey et al. reported overexpression of GLS2 in the luminal subtype of breast cancer. Hence, the exact role of GLS2 in tumorigenesis remains controversial [18].

Analysis of the Cancer Cell Line Encyclopedia (CCLE) project by Yu et al. has revealed that GLS1 plays vital roles in various cancer cell lines. It is upregulated in tumour tissues compared to adjacent normal tissues [19]. Numerous evidence supports the association between overexpressed GLS1 and tumour progression and poor prognosis [19,20], emphasizing the significant role of GLS1 in tumorigenesis and the need to inhibit GLS1 for promising cancer therapy. Lampa et al. have validated the role of GLS using GLS-specific shRNA constructs in TNBC cell lines as well as in animal models [21]. Significant inhibitions of cancer cell growth and decline in downstream metabolite levels were observed upon GLS knockdown, and this was double-checked by α-ketoglutarate (a metabolite downstream of GLS) supplementation, which reverted back the effect caused by GLS knockdown. Similar effects such as decreased tumor growth and alterations in metabolite levels were noted in *in vivo* experiments as well. Downregulated mechanistic target of rapamycin (mTOR) activity and upregulated ATF4 stress response pathway were also observed in responder breast cancer cell lines, when the GLS inhibitor, CB-839, was tested. Moreover, coinhibition of GLS and mTOR was found to be synergistic in responder cell lines, thus highlighting the novel combination strategy for new and potent GLS inhibitors development in basal-type breast cancers, and this study also signifies the adoption of these molecular changes as a predictive cell death protein biomarker during treatment with GLS inhibitor [21]. A recent study by Shiqi et al. has reported the relationship between breast cancer metastasis and glutamine metabolism along with the novel treatment strategies using amino acid transporters and glutaminase as drug targets [22].

### 1.2. The link between glutaminolysis and autophagy in cancer

Glutamine is known to be an effective inhibitor of autophagy, which is an important catabolic mechanism that cells adopt to destroy old and long-lived proteins and organelles [23,24]. The mTOR complex-1 (mTORC1), is the key downstream effector responsible for the inhibition of autophagy. Recent reports document that mTORC1 inhibition provokes autophagy via activating the ULK complex through ULK1/2, ATG13, RB1CC1/FIP200, and ATG101. Activated ULK complex leads to the phosphorylation of BECN1 (a constituent of class III phosphatidylinositol 3-kinase complexes) and subsequently autophagy [25–27]. Following this, the AMPK protein and the TSC1-TSC2 complex direct the growth factors and signaling cascades [28,29]. On the other hand, mTORC1 also inhibits autophagy through cytosolic retention of the transcriptional factor, TFEB, thus stressing the need for mTORC1 inhibition [30]. Even though mTORC1 inhibition stimulates autophagy during nutrient depletion, nutrient repletion reactivates mTORC1 leading to the termination of autophagy.

The first evidence by Mortimore and Schworer proved that amino acids play a vital role in the regulation of autophagy and Blommaart et al. concluded the role of mTOR in mediating the autophagy through amino acids [31,32]. Despite the mechanism behind the correlation between mTORC1 and amino acids is complex and not fully elucidated, recent reports indicate that mTORC1 can identify the presence of glutamine and leucine through glutaminolysis [33]. αKetoglutarate synthesized through glutaminolysis activates mTORC1, by way of increasing the GTP loading of RRAGB (a member of the RRAG family), which translocates mTORC1 to the lysosome surface, and its subsequent activation, thus leading to the inhibition of autophagy [34]. Apart from the role of RRAGB, EGLNs/prolyl hydroxylases play a critical role and constitute a mechanistic link in α-ketoglutarate mediated activation of mTORC1 [35]. However, a recent study highlights the role of acetyl-CoA synthesis and protein acetylation as an alternate mechanism for α-ketoglutarate mediated activation of mTORC1 [36]. A keynote in the inhibitory mechanism of glutaminolysis on autophagy is its byproduct, ammonium, which acts as a doubleedged sword, wherein it triggers autophagy at low concentrations and inhibits at higher concentrations [37].

### 1.3. Association of glutaminase with mTOR activity in cancer

The mTOR pathway acts as an integral regulator of cellular metabolism influencing both intra and extracellular signals. Bar-Peled and Sabatini reported the role of amino acids in controlling the mTOR signaling, whose upregulation is implicated in the pathogenesis of several diseases including cancer [38]. The discovery of a lysosome-based signaling system such as Rags, which is a Rasrelated GTPase along with the Ragulatory v- ATPase, GATOR (GAP activity towards Rags), and folliculin (FLCN) complexes explores the mechanism behind amino acids-induced mTORC1 activation. Recently, glutaminase inhibition was found to downregulate mTOR activity, and suggested that a combination of glutmainase inhibitor with mTOR inhibitor might inhibit cell growth synergistically [21]. Recently, Meng et al. have documented that mTORC1 is activated by glutamine via Rag GTPase–independent mechanism that requires ADPribosylation factor 1 (Arf1) [39]. Duran et al. observed that glutaminolysis tends to trigger the mTOR pathway via altering the GTP binding of RagB in the human sarcoma cells [34]. Other studies prove that mTORC1 stimulates glutamine anaplerosis via activating the enzyme glutamate dehydrogenase, which is implemented by the transcriptional repression of SIRT4 (Sirtuin 4). Further, SIRT4 is suppressed by mTORC1 via provoking the proteasome- mediated destabilization of cAMP response element binding-2. Generally, SIRT4 levels are reduced in cancer conditions, which upon overexpression inhibits cell proliferation, transformation, and tumor development, thus indicating that aiming at nutrient metabolism with upregulated mTORC1 signaling in energy-addicted cancer types may be a prophylactic therapeutic approach [40].

### 1.4. Relationship between glutaminase, mTOR, and autophagy in cancer

Although numerous studies have proved that autophagy is inhibited by mTORC1, which is activated by α-ketoglutarate synthesized from glutamine, the exact role of the bioenergetic status of the cell and its integral regulator behind the relationship of glutamine to mTORC1 is yet unexplored. Bodineau et al. report that AMP activated protein kinase (AMPK) plays a vital role in connecting glutamine and mTORC1 to autophagy [41]. It was further reported that glutaminolysis activation alone can increase the ATP levels and inhibit AMPK (AMP-activated protein kinase), thus implying that the bioenergetic status of the cell is largely influenced by glutaminolysis [41]. Interestingly, it was further observed that glutaminolysis is not required for connecting glutamine to the ATP/AMPK pathway. In the case of glutaminolysis inhibition, the cells drive towards other alternative pathways involving asparagine synthetase (ASNS), glutamateoxaloacetate transaminase and the gamma-amino butyric acid shunt. Previous reports have already connected these key factors to cancer therapy [42]. Nevertheless, Bodineau et al. reported this alternative pathway as an integral pathway in connecting glutamine and mTORC1autophagy signaling. ASNS was observed to metabolize glutamine, which along with glutamateoxaloacetate transaminase and the gamma-amino butyric acid shunt elements provides the entry for the cells to the TCA cycle to synthesize oxaloacetate and ATP synthesis subsequently [41]. Glutaminase and ASNS were also found to balance with each other’s activity upon inhibition of either one of these, thus highlighting the efficiency of glutamine in metabolic homeostasis maintenance and cancer therapy. To note, although leucine also plays a crucial part in mTORC1 activation, its role in mTORC1 signalling is observed only on a short-term basis. However, its activation role can be extended upon the addition of additional amino acids such as glutamine and arginine. Hence, compared to other amino acids, glutamine plays a critical role in α-ketoglutarate production, which is an activator of mTORC1 signalling. Overall, the study throws a controversial conclusion about leucine’s role in mTORC1, while highlighting glutamine as a major amino acid in mTORC1 activation and autophagy, thereon.

**Fig. 1:**
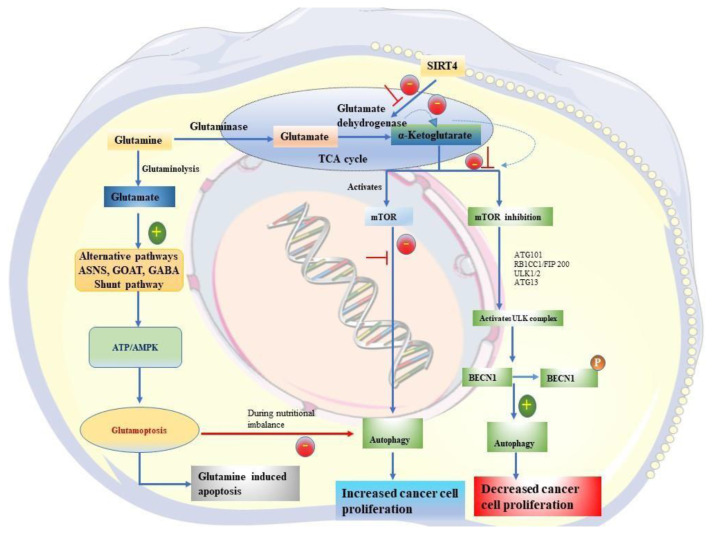
A schematic illustration representing the association between glutaminase, mTOR and autophagy in cancer.

Further, ATP/AMPK has been implicated as a major inducer of glutamoptosis, which refers to glutamine-induced apoptotic cell death by inhibiting autophagy during nutritional imbalance. Hence this study highlights the mechanistic connection between glutaminemTORC1-autophagy, which is exerted through two independent branches such as glutaminolysis and ASNS-GABA shunt pathway [42]. The schematic illustration representing the association between glutaminase, mTOR and autophagy on cancer is given in Fig. 1.

### 1.5. Relationship between glutaminase and mitogen activated protein kinase (MAPK) signaling

A study by Weinberg et al. reported that glutamine catabolism is required for Ki-ras2 Kirsten rat sarcoma viral oncogene homolog (KRAS)-induced cancer cell growth under aerobic conditions [43]. Indeed, reactive oxygen species (ROS) produced during glutamine metabolism are also essential for KRAS-mediated cancer cell growth, via regulation of extracellular signal regulated kinases (ERKs). Numerous evidence indicates the vital part of glutamine in MAPK regulation via, both ERK and c-Jun N-terminal kinases (JNK) signaling in oncogenic cell growth [44,45]. Despite the role of these signaling effects on enhanced tumor cell proliferation and reduced cell death, the exact mechanism is yet unclarified. However, considering the crucial role of glutamine metabolism in signalling through several mitogenic pathways, it is underlined that cell cycle progression is largely influenced by glutamine metabolism. Rhoads et al. have demonstrated that ERK and JNK signalling inhibition decreases activating protein-1 (AP-1)dependent gene transcription during oncogenesis [44]. A study using hepatoma cells delineated that glutamine triggered the expression of sterol regulatory element binding proteins (SREBP) targets, by inducing both SREBP-1a transcription and SREBP processing [46]. Previous reports have suggested that inhibiting glutaminase enzyme has led to a suppressed expression of various sets of genes indispensable for oncogenic growth, which might be due to the alterations in histone methylation [47]. In a recent study by Lee et al. targeting glutaminolysis and associated mitochondrial functions has been highlighted as a potential therapeutic approach for cancer revealing oxidative metabolic vulnerabilities [48]. The above reports are the supporting evidence for the role of glutamine on MAPK signalling in oncogenesis.

### 1.6. Glutaminolysis and redox status in cancer

In general, tumor cells depend on glutamine and hence upregulated glutaminolysis is reported in several cancer types. However, most of the studies focus on the role of glutamine in energy metabolism rather than on its role in maintaining redox homeostasis. Glutamate produced is also used as a precursor for the synthesis of glutathione (GSH), which is an important antioxidant, apart from its flow to Kreb’s cycle [49]. Cancer cells often experience higher levels of oxidative stress due to their rapid growth and altered metabolism. Glutathione helps neutralize ROS and maintain redox balance, allowing cancer cells to survive under stressful conditions.

Therefore, reduction of glutamine through inhibition of GLS1 could decrease GSH levels and enhance the level of oxidative stress leading to apoptotic-mediated cell death in cancer cells [50]. Similarly, Gregory et al. have reported that inhibition of glutaminase using its inhibitor leads to reduced GSH and increased mitochondrial ROS levels, which resulted in apoptosis in several acute myeloid leukaemia cell lines [51]. It has also been proposed that increased GSH levels within cancer cells are associated with enhanced resistance to chemotherapy, and hence selective GSH reduction has been targeted for effective cancer therapy. Further, the study by Gregory et al. also delineated that exogenous supplementation of GSH curbed the abnormal mitochondrial ROS production and apoptosis that was caused by glutaminase inhibition [51]. The above findings strongly support the fact that GSH-dependent redox homeostasis is crucial for cell survival maintenance in several cancer types, including acute myeloid leukaemia.

Several redox-directed therapeutic agents are explored at least as monotherapy for cancer treatment [52,53]. Since glutaminase suppression causes changes in the redox state, studies demonstrate that leukaemia cells might be sensitized through this process to adjuvant pro-oxidant drugs, resulting in enhanced elimination of leukaemia cells through increased cell death [51]. Further, this study documented that glutaminase inhibitor CB-839 when combined with two other Food and Drug Administration-approved leukaemia drugs (inducers of mitochondrial ROS, arsenic trioxide, and homoharringtonine production) reveals significant antileukemic activity in acute myeloid leukaemia under both *in vitro* and *in vivo* conditions. The potential of this combination therapy to remove primary acute myeloid leukaemia colony-forming cells represents the ability of the drug to remove leukaemia stem cells, which is known to depend largely on enhanced mitochondrial metabolism for its survival [54,55]. Gregory et al. have provided evidence that targeting glutamine metabolism in combination with drugs that alters the redox status can act as an prophylactic treatment strategy against solid leukemic tumors [56].

Overall, the above reports stand as evidence for the potential role of glutaminase on redox homeostasis in tumorigenesis.

### 1.7. Inhibition of glutamine uptake by cancer cells via SLC1A5

A report by Wise et al. suggested that SLC1A5, a c-Myc-regulated transporter exerts a key role in arbitrating the glutamine uptake by the cancer cells, and therefore upregulated expression of SLC1A5 was reported in several primary human cancers [6]. Poor prognosis, aggressive biological behavior, and uncontrolled proliferation of glutamine-dependent cancer cells were observed upon upregulated SLC1A5 condition [57]. Inhibition of SLC1A5 by its inhibitor, L-γglutamyl-p-nitroanilide (l-glutamine analog) was found to reduce the uptake of glutamine by the cancer cells via suppressing mTORC1 signaling [57]. Further, lung cancer cell growth and viability were significantly decreased upon targeting SLC1A5, which exerted cell death in part by affecting mTORC1 signaling [58]. To note, Todorova et al. previously reported that potent anticancer drugs such as tamoxifen and raloxifene (selective estrogen receptor modulators) mediate cancer cell death by inhibiting the cellular glutamine uptake by targeting SLC1A5 [59]. The above findings substantiate the potential role of SLC1A5 in stimulating cancer cell death by inhibiting the glutamine uptake of the cancer cells. Likewise, SLC6A14 and SLC38A5 are also important glutamine transporters that mediates the influx of glutamine, serine, glycine and methionine to cancer cells and found to be overexpressed in several cancer types. SLC6A14 is a Na+/Cl- -coupled transporter for multiple amino acids in addition to the above mentioned four amino acids. On the other hand, SLC38A5 is a Na + -coupled transporter with restricted specificity towards glutamine, serine, glycine, and methionine. Importantly, both these transporters are reported to stimulate rapid proliferation of cancer cells [60].

### 1.8. Glutaminase inhibitors and their mechanistic role in clinical trials

To explore the exact role of glutaminase in tumorigenesis, several glutaminase inhibitors such as 6- diazo- 5- oxonorleucine (DON), Bis- 2- (5- phenylacetamido- 1, 3, 4- thiadiazol- 2- yl) ethyl sulphide (BPTES), 5-(3-Bromo-4-(dimethylamino) phenyl)-2,2-dimethyl-2,3,5,6tetrahydobenzo [a] phenanthridine- 4 (1H)-one (Compound 968), JHU-083 and CB-839 have been experimented. Table 1 represents the various glutaminase inhibitors with their mechanistic role. The synthetic compound, DON, is known to inhibit the active site of the glutaminase enzyme irreversibly, and non-specifically binds to various other glutamine-dependent enzymes. Further, the off-target effects of DON are highly toxic to normal cells [61]. Further, to increase the efficiency of DON, its structure was modified by masking the carboxylate and amine functional group, and a prodrug called “JHU-083” was synthesized. The prodrug, JHU-083 was found to inhibit the growth and aggravate the apoptosis of human MYC-expressing medulloblastoma cells [62]. JHU- 083 was also observed to decrease the growth of glioma cells, modulate cellular metabolism, interrupt mTOR signaling, and inhibit cyclin D1 protein expression via glutaminolysis independent mechanism [63]. This study further documented that targeting glutamine metabolism by JHU-083 would be highly efficient in preclinical models of glioma cells. Further, a recent study by Yang et al. have emphasized the drug combination strategies to enhance the efficiency of inhibitors of glutamine metabolism as cancer therapeutics [64].

The other inhibitors, BPTES and compound 968 are allosteric inhibitors that do not compete for the active site with glutamine. BPTES maintains the enzyme in a non-active tetrameric state, independent of inorganic phosphate (Pi), while compound 968 binds to GLS1 isoform in an inactive state and prevents the active tetrameric formation in the absence of Pi [65,66]. Compound 968 is a GLS2 inhibitor, prevents GLS2 mediated anaplerosis, and is more potent against BPTES-resistant cell proliferation and tumorigenesis in breast cancer. Despite their potency, BPTES and compound 968 exhibit varied benefits, specificity, and bioavailability in clinical trials, demanding the need for new GLS inhibitors with enhanced specificity and ability. On the other hand, CB-839, another potent, selective, and orally bioavailable GLS1 inhibitor is currently used in clinical trials against various types of cancers [13]. In the case of breast cancer, CB-839 demonstrated antiproliferative activity in triplenegative breast cancer cells and no antiproliferative effect in estrogen-positive cells. Further, CB-839 was found to be effective both as a single agent as well as in combination with paclitaxel. Several other GLS inhibitors such as ebselen, chelerythrine, and apomorphine were explored, which revealed 101,500-fold higher affinity and about 100-fold enhanced inhibitory efficiency as compared to DON and BPTES [67]. Despite the efficiency of these currently available inhibitors, the lack of specificity extends the search for novel lead molecules that can be more potent in cancer therapy.

### 1.9. Targeting glutaminase in cancer-friend or foe?

Glutamine metabolism is targeted as a prophylactic approach in cancer treatment, and indeed, it exhibits a potent effect in reducing cancer progression. However, the other side of inhibiting glutamine metabolism should be considered definitely, as glutamine is required for immune regulation and the proliferation of lymphocytes, macrophages, and neutrophils in normal cells [68,69]. Alterations in glutaminase activity can impact the immunosuppressive tumor microenvironment, potentially affecting antitumor immune responses [70]. It has been reported that glutamine is required by both immune cells and tumor cells for their survival, proliferation and function. In case of immune cells, reprogramming of glutamine metabolism affects their phenotypes, which makes them to act as either pro-tumorigenic or anti-tumorigenic agent. On the other hand, reprogramming of glutamine metabolism in the tumor cells favors their uncontrolled proliferation and survival in a hypoxic tumor microenvironment. Overall, the above observation concludes that there exist a significant association in glutamine metabolism between immune cells and tumor cells in the tumor microenvironment. Gastrointestinal and neurotoxicity were noted during the early trials conducted using I-DON, a glutamine mimetic anti-metabolite, as glutamate is required as a neurotransmitter in neurons, which is then converted back to glutamine for transporting back to neurons [71]. The above study on Gls-null and Glsheterozygous mice indicates the importance of glutaminase in the central nervous system, by observing the death of animals due to insufficient glutamate. Furthermore, the glutaminase-null mice were also observed to induce respiratory acidosis in a study carried out by Masson et al. [72]. Importantly, during metabolic acidosis, kidney acts as the major organ of glutamine extraction and catabolism. This enhanced renal extraction of glutamine is balanced by decreased utilization in the intestine and increased release from muscle and liver. However, in case of chronic acidosis, the above mechanism continues in part by enhanced expression of renal genes that encode key enzymes of glutamine metabolism and various transport proteins, thus highlighting the role of glutamine in the normal functioning of kidney [73]. Considering the above reports, it should be noted that glutaminase role is indispensable for normal energy metabolism and organ functions. On the other hand, inhibiting glutaminase helps prevent cancer progression to a greater extent, despite affecting the energy demand of the normal cell. Hence, an appropriate dosage level of glutaminase inhibitor should be adopted during cancer conditions, and it also paves the way for the need for further research for careful glutaminase inhibition.

## 2. Conclusion

The present review highlights the mechanistic role of glutaminase in cancer conditions and the advantages of targeting glutaminase by glutaminase inhibitors in cancer treatment. The positive physiological role of glutaminase in a normal cell is also emphasized to employ the inhibitors that hold the potential to target only the cancer cells. In conclusion, the present review emphasizes the pitfalls and resolutions of various inhibitors that are currently under clinical trials, and forces the need for novel lead molecules to safely and specifically target glutaminase in cancer therapy.

## Figures and Tables

**Table 1:** Mechanistic role of various glutaminase inhibitors.

S.No	Inhibitor	Role	References
1.	6-diazo- 5-oxonorleucine (DON)	Inhibits the active site of the glutaminase enzyme irreversibly, and binds to various other glutamine-dependent enzymes, nonspecifically	[60]
2.	Bis-2-(5-phenylacetamido-1, 3, 4- thiadiazol- 2- yl) ethyl sulphide (BPTES)	BPTES is an allosteric inhibitor that do not compete for the active site with glutamine. BPTES maintains the enzyme in a non-active tetrameric state.	[64,65]
3.	5-(3-Bromo-4-(dimethylamino) phenyl)-2,2-dimethyl-2,3,5,6tetrahydobenzo [a] phenanthridine-4 (1H)-one (Compound 968)	It is a GLS2 inhibitor that prevents GLS2 mediated anaplerosis, and is more potent against BPTESresistant cell proliferation and tumorigenesis	[65]
4.	JHU-083	Structurally modified version of DON. It modulates cellular metabolism, interrupt mTOR signaling, and inhibit cyclin D1 protein expression via glutaminolysis independent mechanism. It inhibits the growth and aggravate the apoptosis of cancer cells.	[61,62]
5.	CB-839	It is a selective and orally bioavailable GLS1 inhibitor that is currently used in clinical trials against various types of cancers	[13]
